# Bullied Because of Their Teeth: Evidence from a Longitudinal Study on the Impact of Oral Health on Bullying Victimization among Australian Indigenous Children

**DOI:** 10.3390/ijerph19094995

**Published:** 2022-04-20

**Authors:** Md Irteja Islam, Verity Chadwick, Tuguy Esgin, Alexandra Martiniuk

**Affiliations:** 1 Sydney School of Public Health, Faculty of Medicine and Health, The University of Sydney, Edward Ford Building, A27 Fisher Road, Sydney, NSW 2006, Australia; alexandra.martiniuk@sydney.edu.au; 2 Centre for Health Research, School of Business, The University of Southern Queensland, West Street, Darling Heights, Toowoomba, QLD 4350, Australia; 3 Royal North Shore Hospital, Reserve Rd., St. Leonards, Sydney, NSW 2065, Australia; veritychadwickjackman@gmail.com; 4 Discipline of Exercise and Sports Science, Faculty of Medicine and Health, The University of Sydney, Level 6 Susan Wakil Health Building D18, Western Ave, Camperdown, Sydney, NSW 2050, Australia; tuguy.esgin@sydney.edu.au; 5 School of Medical and Health Sciences, Edith Cowan University, 270 Joondalup Drive, Joondalup, WA 6027, Australia; 6 School of Management and Governance UNSW Business School, University of New South Wales, Kensington, Sydney, NSW 2052, Australia; 7 Office of the Chief Scientist, The George Institute for Global Health, Level 5/1 King Street, Newtown, Sydney, NSW 2042, Australia; 8 Dalla Lana School of Public Health, The University of Toronto, 155 College St. Room 500, Toronto, ON M5T 3M7, Canada

**Keywords:** bullying, oral health, indigenous, children, Australia

## Abstract

Making life better for Indigenous peoples is a global priority. Although bullying and oral health have always been a topic of concern, there is limited information regarding the impact of this problem on the general population, with no evidence in this regard among the Australian Indigenous population. Thus, we aimed to quantify the relationship between bullying victimization and oral health problems by remoteness among 766 Australian Indigenous children aged between 10–15-years using data from the LSIC study. Bivariate and multilevel mixed-effect logistic regression analyses were employed. Findings indicated children self-reported bullying more than parents reported their children were being bullied (44% vs. 33.6%), with a higher percentage from rural/remote areas than urban areas. Parents reported that oral health problems increased the probability (OR 2.20, *p* < 0.05) of being bullied, in Indigenous children living in urban areas. Racial discrimination, lower level of parental education and poor child oral hygiene increase the risk of bullying victimization. Parental happiness with life and a safe community were associated with a lower risk of bullying. Dental problems are linked with Australian Indigenous children experiencing bullying victimization. Cultural resilience and eliminating discrimination may be two modifiable paths to ameliorating health issues associated with bullying in the Australian Indigenous community.

## 1. Introduction

In Australia, Aboriginal and Torres Strait Islander peoples (respectfully referred to hereafter as Indigenous) are flourishing. Many initiatives focus on empowering communities by engaging with local knowledge and influence [1], and these collaborations between researchers and communities are not only working to achieve desired outcomes but also serve as exemplars of reconciliation. While these working relationships between communities, researchers and policymakers have not yet resulted in all the desired socio-emotional well-being outcomes, such relationships work to ensure Indigenous communities’ involvement, participation, ownership and endorsement of the implemented programs.

According to the most recent National Strategic Framework for Indigenous Australians [2], a positive sense of social and emotional wellbeing (SEWB) is important for the overall health status (both physical and mental) of Indigenous peoples’ although SEWB might vary across the course of their life. In broad terms, SEWB is a holistic model resulting from a web of interactions between individuals, families, kin, and the community. It also recognizes the significance of an individual’s connection to land, culture, religion, and history, as well as how these affect the individual [3]. Previous studies indicate different variables affect children’s SEWB compared to their parents [2,4]. Miller et al. affirmed this by identifying some important factors that parents believe are attributable to Indigenous children’s health and social outcome [5]. Moreover, evidence suggests since colonisation, many health issues have disproportionately affected Australian Indigenous adolescents, and dental problems are one of these [6,7,8,9,10]. For instance, approximately 6 out of 10 Indigenous Australian children report dental decay and periodontal disease [10,11]. Moreover, children with dental problems are more likely to live in rural/remote Australia and be from low-income families, contributing to geographical and financial access barriers to dental care [12]. Poor oral health causes pain and disfigurement and negatively influences the quality of life and general life satisfaction, and is associated with poor nutrition, diabetes, and cardiovascular disease [13,14,15]. In general, poor dentition and poor oral health have also been linked to bullying in both children and adults [16,17,18].

Physical characteristics, including dentition, are remarkably important among adolescents, with attractiveness conveying high status in many group settings and playing a role in fitting in [19]. Therefore, deviations in ‘normal’ dentition can lead to bullying victimisation [17,20,21,22,23,24]. It is reported that children who had oversized front teeth, missing teeth, extra spaces between them, or coloured teeth with unusual shapes are more likely to be bullied [25]. Bullying can be physical, social, or psychological but it always involves unprovoked intent to harm [26]. Students who are bullied tend to suffer from poorer health [27], lower self-esteem [28], more somatic complaints [29], interpersonal difficulties [30], higher levels of loneliness [31], depression and suicidal ideation, and anxiety [32]. Children who are bullied are more likely to both dislike [33] and want to avoid school [34], so their academic achievement tends to be lower, and levels of absenteeism tend to be higher [35]. Evidence suggests children from culturally and linguistically diverse backgrounds, socioeconomically disadvantaged schools and children living in rural/remote areas are more likely to be bullied compared to their counterparts [11,36,37]. Protective factors against childhood bullying include positive family characteristics (e.g., family functioning, family structure), good parental health and wellbeing, and above average motor skills [38,39]. In addition, Indigenous culture reputed to have a protective effect in building strong families and safe communities for Indigenous children [40]. In Australia, the prevalence of childhood bullying varies. For example, a longitudinal study shows that 7 out of 10 non-Indigenous Australian aged 12–13 years had experienced bullying in the past-12 months [41], while another study reported about 56% of children aged 10 years were bullied monthly in a year [42]. 

Despite the recent increasing interest of researchers and clinicians in the psychological and functional consequences of poor oral health, little is known about the impact of oral health on bullying in children and adolescents [25,43]. Further, a recently published systematic review acknowledged the association between poor dental health and bullying is controversial among school children and adolescents [42]. While, some Indigenous Australians have reported both bullying [26] and poor oral health [44,45,46] as ‘normal’ problems to have. A few initiatives are aiming to improve these issues among Australian general populations, although separately e.g., [47,48]. Moreover, to the best of our knowledge, no study has investigated the link between poor oral health and bullying victimization in Australian Indigenous children.

Therefore, this study aims to help close the quantitative evidence gap about the contribution of oral health, and other sociodemographic variables, to the well-being of Australian Indigenous children. We first aimed to estimate the frequency of dental problems and bullying victimisation by area of location (rural/remote and urban. Secondly, we sought to explore if a relationship between bullying and poor oral health exists (stratified by remoteness) among Indigenous children aged 10–15 years using the latest survey data from the Longitudinal Study of Indigenous Children (LSIC). 

## 2. Materials and Methods

This paper is being reported according to STROBE guidelines for observational studies [49].

### 2.1. Study Design

Footprints in Time: The Longitudinal Study of Indigenous Children (LSIC), is the first large-scale prospective cohort study to focus on the strengths and challenges experienced by Australian Indigenous children and families [50,51]. Extensive community engagement is one of the key components of the LSIC, as is the leadership, consisting of a steering committee that is predominantly Indigenous people [50,52]. 

The LSIC team originally recruited two cohorts of Indigenous children—the younger (B) cohort (aged 0.5–2.0 years) and the older (K) cohort (aged 3.5–5.0 years) at a baseline survey in 2008 (termed Wave 1). A non-random, purposive sampling design, with 11 clusters were chosen by the LSIC to be broadly representative of the socioeconomic and community contexts where Indigenous children live [50,53]. A total of 1671 children were enrolled at Wave 1 and were involved in the following waves where possible. The sample retention was 88% from previous waves at Wave 11 (the most current wave available for analysis) in 2018 [52]. Data were collected annually via face-to-face interviews between an Indigenous interviewer and the respondent (study child, parent or primary caregiver). Each eligible family was contacted, and voluntary written informed consent was obtained from parents/caregivers for their children [52]. Ethical approval has been obtained from the Human Research Ethics Committees of the Australian Government Department of Health and each state, territory, or region. More details regarding the study design and data collection procedure have been reported elsewhere [50,52,54]. 

### 2.2. Participants

**Figure 1:**
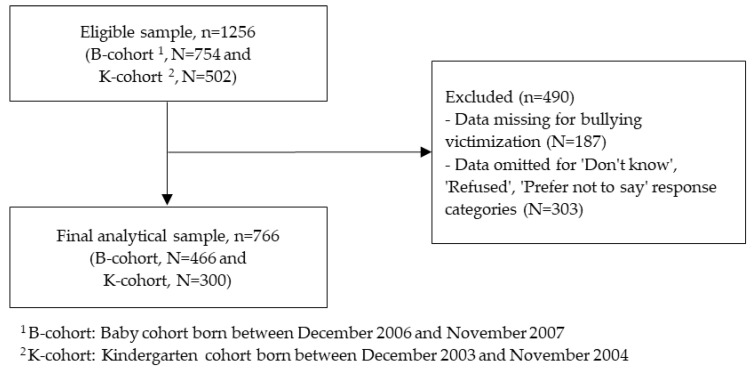
Flow diagram showing the selection of samples in the final analysis.

The current study included 1256 Indigenous children aged 10–15 years at the time of Wave 11 of the LSIC in 2018. Out of 1256 children, 754 and 502 children were from B-cohort and K-cohort, respectively. Figure 1 shows the flow diagram of the final analytical sample (*n* = 766). Participants included in our study are those who have completed data on the outcome variable (i.e., bullying victimization) and main exposure variable (i.e., oral health) obtained from the parent questionnaire and study child questionnaire. The categories of ‘Don’t know’, ‘Prefer not to say’ and ‘Refused’ responses were omitted. A complete case analysis (CCA) was performed since data were missing completely at random and were representative of the entire study sample. Moreover, CCA typically produces unbiased results in regression models as it uses actual data, rather than simulated data [55].

### 2.3. Measures

Evidence from the literature informed our selection of variables for the study. Previous studies indicated predisposing factors (e.g., age, gender, schooling, parental education and occupation, racism), enabling factors (e.g., remoteness, family income, socioeconomic status), strength-based protective factors (e.g., family functioning, personal wellness and wellbeing, community safety), as key features related to mental health among Indigenous populations [56,57,58]. The health variables of interest were dental and oral health problems. The measures used are summarized in Table 1. 

### 2.4. Data Analyses

Initially, the characteristics of the sample (*n* = 766) from Wave 11 of the LSIC were outlined by descriptive statistics in the form of frequency (*n*) and percentages (%), according to the Australian Statistical Geography Standard (ASGC) Remoteness area across categories of explanatory sociodemographic variables. Then, Pearson’s Chi-squared tests were deployed to examine the bivariate associations between each explanatory variable and the main outcome variable. Following this, as recommended by the LSIC research team, multilevel logistic models were used to examine the association between explanatory variables (oral health and other covariates) and outcome variables (bullying victimization by remoteness) [54]. Predictors were included in the adjusted model only if a covariate was statistically significant (*p* < 0.05) in the bivariate analyses. We used multilevel mixed-effect logistic regression models as it adjusts parameter estimates and standard error for the clustering of study participants within a geographical area. We use Stata/SE 14.1 for all analyses and report adjusted odds ratios (aOR) with 95% confidence intervals (95% CI) for each model.

## 3. Results

### 3.1. Sample Characteristics

Characteristics of the final sample by remoteness are shown in Table 2. In this study, more than two-thirds of Indigenous children (67.4%) were living in the rural/remote areas, and 32.6% were in the major cities. Overall, there was a high proportion of children aged between 10–≤12 years compared to the ≥13–15 years age-group, but distributions for gender were similar. More than 50% of children were enrolled in Grade 5 and Grade 6 with a higher proportion in rural/remote areas, and 83% of children attended a government school for education. Most parents (about 50%) had completed Year-12/below with a higher percentage in rural/remote areas (71.7%) than in major cities (28.4%), while the proportion of employed parents was higher in rural/remote areas (64.1%) compared to those living in major cities (35.9%). Table 2 also shows a similar pattern for parents’ happiness with life, safe community and good family functioning with percentages around 32% in cities and 68% in rural/remote areas for each category, respectively. Unfortunately, almost 49% of the Indigenous families had experienced racial discrimination, where 40% of them were living in major cities and about 40% were in the rural/remote areas. Most of the families had a medium to high income per fortnight, but according to SEIFA IRSAD quintiles, a higher percentage of families (more than 72%) were from the most disadvantaged groups (Q1–Q2) compared to advantaged groups (Q3–Q5) in Table 2.

### 3.2. Prevalence of Bullying and Dental Problems

**Figure 2:**
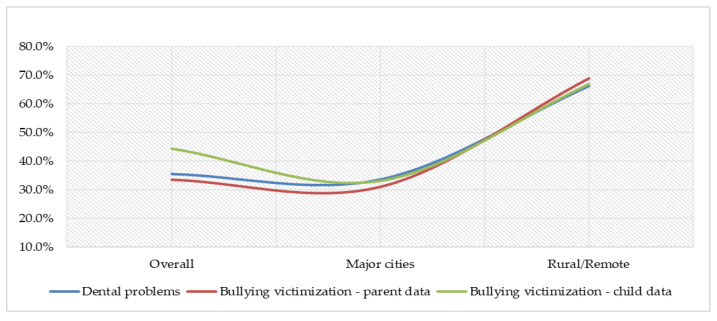
Prevalence (%) of dental problem and bullying victimization.

Overall, 273 (35.6%) Indigenous children had any dental problems, 92 (33.7%) from major cities and 181 (66.3%) from rural/remote areas (Figure 2). Of the total respondents, the percentages of being bullied were higher when reported by children themselves compared to reporting by the parent (44.4% vs. 33.6%). Figure 2 also depicts that bullying victimization was more prevalent among those who were living in rural/remote areas compared to those who were living in major cities.

### 3.3. Bivariate Relationships between Explanatory Variables and Bullying Victimization

As parents reported in Table 3, 47.5% (*p* < 0.05) of bullied children living in major cities had dental problems, while the percentage was 40.7% (*p* < 0.05) in rural/remote areas. More than 70% of parents of bullied children were satisfied with their life in major cities compared to those who reported neither or were unhappy. Bullied children living either in major cities or in rural/remote areas felt they had safe communities but unfortunately still experienced racism (*p* < 0.05 for all). 

Child-reported data in Table 4 shows that Indigenous children living in cities with dental problems experience bullying victimization (*p* < 0.05). Age and parental happiness were associated with this bullying victimization for children living in major cities. While bullied children living in rural/remote areas were associated with age, feelings regarding the community being unsafe and oral hygiene practices (*p* < 0.05 for all).

### 3.4. Logistic Models for the Odds of Bullying Victimization

In Table 5, the results of the logistic regression models are presented. These models were used to investigate the association between bullying victimization, and dental problems and other potential covariates. Parent data shows that children who had any dental problem were 2.20 times (95% CI, 1.20–4.02) more likely to be bullied in major cities than those who had no dental problems. Both parent data and child data demonstrate that children with parents with lower levels of education (who completed Certification III/IV) were significantly associated with bullying in only rural/remote areas compared to those parents with higher education (who completed Year12 or Diploma). 

Children who were living in unsafe communities in rural/remote areas were 2.65 times (95% CI, 1.30–5.40) [parent data] and 2.44 times (95% CI, 1.41–3.86) [child data] more likely to experience bullying victimization than those who had a safe community. Only child data found that children with poor oral hygiene were significantly (OR 2.33, 95% CI, 1.41–3.86) associated with bullying in rural/remote areas. In contrast, from the parent-reported data, bullying victimization was significantly associated with families who experienced racism regardless of location (major cities: OR 3.27, 95% CI, 1.70–6.29; rural/remote: OR 2.39, 95% CI, 1.58–3.63) compared to their counterparts. 

## 4. Discussion

This study examines the relationship between poor dental health and the bullying experience of Australian Indigenous children. Our findings indicate that both bullying and poor dental health were present in major cities and in rural/remote Australia, however, poor dental health was only significantly associated with bullying victimisation in major cities, and not in rural/remote Australia. Parental happiness with life which is an important component of SEWB [11,59] was a protective factor regarding whether a child experienced bullying, while the family experience of racial discrimination or prejudice was associated with bullying victimisation. This study highlights the importance of providing equitable access to dental healthcare in Australia including culturally appropriate services, and the importance of strengthening Indigenous SEWB for the benefit of the community.

Our study found poorer oral health was significantly associated with a higher likelihood of being bullied in urban areas for Indigenous adolescents. We hypothesise that this bullying due to poor oral health in urban areas may be more pronounced than in rural areas possibly because parents cannot afford private dental services for their children or may not have access to, or may be unaware of, or are waiting for, public dental services. This lack of access to dental services may result in Indigenous children having poor dental health (e.g., dental anomalies, mouth pain, and bad breath), and consequently, this may increase their chances of being bullied [17,19,24]. This may be in comparison to their non-Indigenous peers who may be more likely to access dental care and may, therefore, have better oral health.

Research suggests that a smile denotes self-esteem, self-confidence and well-being [61] and children with concerns about their teeth tend to smile less [62]. A recent survey reported that 30% of Indigenous Australians did not access services (including dental services) over the past-12 months when needed. Of these, 32% did not access the dentist primarily due to cost [63,64]. One urban Australian study found some parents avoided public clinics for preventive dental healthcare because of shame about the state of their children’s teeth, negative experiences from the services such as dentists wrongly reporting families to social services, and other concerns superseding prophylactic care [65]. Parents commonly reported that dentists in public clinics would remove teeth rather than provide restorative services [65], further exacerbating the difference in appearance between Indigenous children and their urban peers. This has been echoed by other studies [66,67,68,69], in addition to concerns including long waiting lists, inaccessibility of transport, cultural safety and discrimination against Indigenous families for the state of their oral health, for being Aboriginal, or stigma surrounding the service [65]. Moreover, evidence suggests that there is still a large gap between the dental health of Indigenous and non-Indigenous Australians in urban areas, as dental health care has not been culturally tailored [70].

Our study also revealed that although a high proportion of total respondents in rural/remote areas experienced bullying and/or poor dental health, the proportion of bullied victims with dental problems was low in rural/remote areas compared to urban areas. Moreover, we found that the association between dental problems and bullying in rural areas was not statistically significant, which is inconsistent with previous studies. For example, Coffin reported a very high level of bullying among rural Indigenous youths with 40% of respondents saying they saw or experienced bullying every day or nearly every day [26]. Higher levels of bullying in rural communities have been partly due to a lack of access to employment, transport, and services, such as mental health support [26], and particularly in impoverished rural areas, bullying is often ignored by teachers [26,71]. For Indigenous Australians, the higher rate of bullying in rural/remote areas is compounded by the historical context of colonisation and subsequent policies of assimilation.

Good parental SEWB significantly reduced the probability of children being bullied, as well as poor dental health in major cities. Bullying was more likely to be experienced by children in families where the parents had experienced racism. Happy parents are more likely to be able to emotionally support their children [72,73] and establish strong cultural connections in the community [74], both of which are known to be protective against bullying [75]. High SEWB may also lead parents to encourage good oral hygiene and seek dental health care for their children even if parents are uncomfortable with the services offered. Furthermore, high SEWB may be associated with higher levels of employment [76,77,78] potentially overcoming the barrier of cost when accessing private healthcare.

Racial discrimination experienced by families may be associated with increased rates of bullying and poor dental health in the same way that discrimination is associated with poor mental health [66,79]. Indigenous children experience some of the highest levels of marginalisation in the world [66,80,81]. Experiences of racial discrimination may lead to parents not seeking dental healthcare for their children or waiting until only removing teeth is viable, and this may lead to bullying in school [17,20,21,22,23,82,83].

Bullying in school has significant implications and must be addressed given its prevalence among Indigenous adolescents. Victims of bullying are less likely to finish school, have poorer mental health [75,84,85], have greater internalising and externalising problems [86], and are more likely to use alcohol and marijuana than non-victims [87]. Coffin notes that schoolyard bullying is described by Aboriginal students as “something that Aboriginal people do” p. 90, and is affected by the “wider socioeconomic context which, in this case, has been shaped by centuries of institutional racism toward Aboriginal Australians” p. 85 [26]. This indicates reducing the prevalence of bullying among Indigenous adolescents may be difficult.

This study was the first, to the best of our knowledge, finding that Australian Indigenous children with poor dentition experience higher rates of bullying. These findings contribute to our understanding of the relationships between poor dental health and bullying for Indigenous children in both urban and rural/remote areas and highlight potential protective factors. The LSIC data has a fairly large sample, and the sites selected were intended to reflect the geographic distribution of Indigenous children within Australia and their varied environments [88]. However, our study has some limitations. The sample of Indigenous children is, despite best efforts, not nationally representative, and therefore, may not be generalizable for non-Indigenous Australian children. The data was cross-sectional, and therefore, cannot conclude with certainty a causal relationship. However, evidence suggests that in Aboriginal populations bullying for appearance differences occurs as it also does with other populations [26]. In addition, we were restricted only to include school-bullying in general as the other forms of bullying, such as cyberbullying and social isolation were not available in the dataset. Moreover, we could not include some important factors, such as performance and behaviours at school, physical activity and sporting behaviours of Indigenous children as these data were not collected in the survey that we used in this study, though these variables have been proposed predictors of bullying behaviours in prior studies [38,89]. Lastly, considering the focus is on oral health and bullying, the analytical methods used may lead to selection bias in the results. Further, self-reported data may result in recall bias and social-desirability bias, although past studies validated that self-reporting is the most credible method for determining children’s and adolescents’ health risk behaviours [90,91].

## 5. Conclusions

This study generates new evidence surrounding the relationship between oral health and bullying in a cohort of Australian Indigenous children. The proportion of participants reporting experiencing bullying and poor oral health is unacceptably high in this sample. Racism and poor oral hygiene increase the likelihood of being bullied. Further, our study identifies two protective factors against bullying for Indigenous adolescents. These factors are strong parental social and emotional well-being (SEWB) and living in a community that is self-reported as being safe. These findings suggest future efforts aiming to decrease bullying against Indigenous adolescents should be directed at strengthening the communities, reinforcing positive behaviours, and enhancing cultural resilience to reduce disparities, as well as to promote health and wellbeing among our Indigenous population.

## Figures and Tables

**Table 1:** List of variables.

Variables	Longitudinal Study of Indigenous Children (LSIC) Interview Question Wording or Description	Data Source	Coding for Analysis
Outcome variables			
Bullying victimization	Has the study child been bullied (or treated unfairly) at school (in the previous 12 months)?	Both—parent and child questionnaire	1 = Yes, 0 = No
Remoteness	Australian Statistical Geography Standard (ASGC) Remoteness area 2016	Both—parent and child questionnaire	1 = Major cities, 0 = Rural/Remote
Main explanatory variables			
Dental problems	Has the study child ever had any of the following problems with (his/her) teeth or gums? - Any cavities, holes, or tooth decay - Teeth pulled out because of decay - Abscesses or inflammation - Pain, swelling for more than one week - Bleeding gums - Accident-causing breakage or loss - Overcrowding, needs braces/plate/retainer - Any other teeth problems	Parent questionnaire	1 = Yes, 0 = No
Sociodemographic covariates			
Age	Age of study child (in years)	Both—parent and child questionnaire	1 = ≥13 to 15, 0 = 10 to ≤12
Gender	Gender of the study child	Both—parent and child questionnaire	1 = Boys, 0 = Girls
School grade	In what year/grade is the study child currently enrolled?	Both—parent and child questionnaire	1 = Yes, 0 = No
School type	What kind of school does study child go to?	Parent questionnaire	2 = Government, 1 = Catholic, 0 = Private
Parents’ educational qualification	What was the highest qualification that you have completed?	Parent questionnaire	2 = Diploma and above, 1 = Certificate III or IV, 0 = Year 12 and below
Parents’ employment status	Do you have a job?	Parent questionnaire	1 = Employed, 0 = Unemployed
Parents’ satisfaction with life	How much do you agree or disagree with the following statement? In general, I am happy with how things are for me in my life right now. Note that ‘overall satisfaction with life’ is a direct indicator of SEWB across different populations—children, young people and adults [59]	Parent questionnaire	2 = Unhappy, 1 = Neither, 0 = Happy
Community safety	Do you (parent and child asked separately) think where you live/in your community that is safe during the day and at night?	Both—parent and child questionnaire	1 = Safe, 0 = Unsafe
Child oral hygiene practice	How often are study child’s teeth cleaned?	Both—parent and child questionnaire	1 = Rarely/never, 0 = Once a day or more
Family functioning	Do your family get along well with each other? Note that ‘family functioning’ is multi-component factor (includes cohesion, safety at home and perceived parental support), and good family functioning found to be protective against school bullying [60].	Both—parent and child questionnaire	1 = Very good/Good, 0 = Fair/Poor
Family experiences racial discrimination	How often does your family experience racism, discrimination, or prejudice?	Both—parent and child questionnaire	1 = Frequently, 0 = Occasionally/Never
Family income	How much money do you usually get from all of your sources of income in total, (including your partner), after deductions are taken out, such as tax, quarantined payments, etc.?	Parent questionnaire	2 = High ($2000 or more per fortnight), 1 = Medium ($800–$1999 per fortnight), 0 = Low ($0–$799 per fortnight)
SEIFA IRSAD quintiles	The SEIFA (Socio-Economic Indexes for Areas) IRSAD (Index of Relative Socio-economic Advantage and Disadvantage) is used to estimate area-level SES. A lowest IRSAD score (Quintile 1, 0–20%) signifies greater disadvantage, as well as a lack of advantages in general and highest IRSAD score (Quintile 5, 80–100%), indicates greater advantages as well as a lack of disadvantage at the area level.	Parent questionnaire	4 = Q5 (Most advantaged), 3 = Q4, 2 = Q3, 1 = Q2, 0 = Q1 (Most disadvantaged)

**Table 2:** Sample characteristics.

	Total	Major Cities	Rural/Remote
	*n* (%)	*n* (%)	*n* (%)
Total	766 (100.0)	250 (32.6)	516 (67.4)
Age (Mean = 12.25, SD = 1.49)			
10 to ≤12	466 (60.8)	152 (32.6)	314 (67.4)
>12 to 15	300 (39.2)	98 (32.7)	202 (67.3)
Gender			
Boys	381 (49.7)	122 (32.0)	259 (68.0)
Girls	385 (50.3)	128 (33.3)	257 (66.7)
School Grade			
Grade 4	15 (2.0)	4 (26.7)	11 (73.3)
Grade 5	232 (30.3)	74 (31.9)	158 (68.1)
Grade 6	201 (26.2)	71 (35.3)	130 (64.7)
Grade 7	34 (4.4)	6 (17.7)	28 (82.3)
Grade 8	184 (24.0)	60 (32.6)	124 (67.4)
Grade 9	100 (13.1)	35 (35.0)	65 (65.0)
School type			
Private	52 (6.8)	25 (48.1)	27 (51.9)
Catholic	76 (9.9)	33 (43.4)	43 (56.6)
Government	638 (83.3)	192 (30.1)	446 (69.9)
Parents’ educational qualification			
Year 12 or below	381 (49.7)	108 (28.4)	273 (71.7)
Certificate III or IV	240 (31.3)	73 (30.4)	167 (69.6)
Diploma or above	145 (18.9)	69 (47.6)	76 (52.4)
Parents’ employment status			
Unemployed	315 (41.1)	88 (27.9)	227 (72.1)
Employed	451 (58.9)	162 (35.9)	289 (64.1)
Parents’ satisfaction with life			
Unhappy	63 (8.2)	24 (38.1)	39 (61.9)
Neither	78 (10.2)	28 (35.9)	50 (64.1)
Happy	625 (81.6)	198 (31.7)	427 (68.3)
Community safety			
Safe	704 (91.9)	230 (32.7)	474 (67.3)
Unsafe	62 (8.1)	20 (32.3)	42 (67.7)
Child oral hygiene practice			
Once a day or more	637 (83.2)	213 (33.4)	424 (66.6)
Rarely or never	129 (16.8)	37 (28.7)	92 (71.3)
Family functioning			
Fair/Poor	20 (2.6)	6 (30.0)	14 (70.0)
Very good/Good	746 (97.4)	244 (32.7)	502 (67.3)
Family experiences racial discrimination			
No	392 (51.2)	97 (24.7)	295 (75.3)
Yes	374 (48.8)	153 (40.9)	221 (59.1)
Family income			
Low ($0–$799 per fortnight)	75 (9.8)	22 (29.3)	53 (70.7)
Medium ($800–$1999 per fortnight)	348 (45.4)	97 (27.9)	251 (72.1)
High ($2000 or more per fortnight)	343 (44.8)	131 (38.2)	212 (61.8)
SEIFA IRSAD quintiles			
Q1- Most disadvantaged	371 (48.4)	91 (24.5)	280 (75.5)
Q2	182 (23.8)	51 (28.0)	131 (72.0)
Q3	114 (14.9)	51 (44.7)	63 (55.3)
Q4	65 (8.5)	34 (52.3)	31 (47.7)
Q5—Most advantaged	34 (4.4)	23 (67.7)	11 (32.3)

**Table 3:** Bivariate associations between explanatory variables and bullying victimization by remoteness—Parent data.

	Bullying Victimization—Parent Data			
	Major Cities (*n* = 80)		Rural/Remote (*n* = 177)	
	*n* (%)	*p*-Value	*n* (%)	*p*-Value
Dental problems		0.016 *		0.050 *
No	42 (52.5)		105 (59.3)	
Yes	38 (47.5)		72 (40.7)	
Age		0.859		0.415
10 to ≤12	48 (60.0)		112 (63.3)	
>12 to 15	32 (40.0)		65 (36.7)	
Gender		0.422		0.689
Boys	42 (52.5)		91 (51.4)	
Girls	38 (47.5)		86 (48.6)	
School Grade		0.931		0.629
Grade 4	1 (1.2)		2 (1.1)	
Grade 5	22 (27.5)		57 (32.2)	
Grade 6	25 (31.3)		49 (27.7)	
Grade 7	1 (1.2)		7 (4.0)	
Grade 8	19 (23.8)		40 (22.6)	
Grade 9	12 (15.0)		22 (12.4)	
School type		0.735		0.812
Private	9 (11.3)		10 (5.7)	
Catholic	12 (15.0)		13 (7.3)	
Government	59 (73.7)		154 (87.0)	
Parents’ educational qualification		0.872		0.061
Year 12 or below	33 (30.6)		81 (29.7)	
Certificate III or IV	25 (34.3)		67 (40.1)	
Diploma or above	22 (31.9)		29 (38.2)	
Parents’ employment status		0.601		0.871
Unemployed	30 (37.5)		77 (43.5)	
Employed	50 (62.5)		100 (56.5)	
Parents’ happiness with life		0.014 *		0.619
Unhappy	14 (17.5)		12 (6.8)	
Neither	9 (11.3)		20 (11.3)	
Happy	57 (71.2)		145 (81.9)	
Community safety		0.022 *		0.010 *
Safe	69 (86.3)		155 (87.6)	
Unsafe	11 (13.7)		22 (12.4)	
Oral hygiene practice		0.061		0.384
Once a day or more	64 (80.0)		144 (79.7)	
Rarely or never	16 (20.0)		33 (20.3)	
Family functioning		0.415		0.068
Fair/Poor	1 (1.3)		8 (4.5)	
Very good/Good	79 (98.7)		169 (95.5)	
Family experiences racism		<0.001 ***		<0.001 ***
No	17 (21.3)		77 (43.5)	
Yes	63 (78.8)		100 (56.5)	
Family income		0.096		0.324
Low ($0–$799 per fortnight)	11 (13.7)		23 (13.0)	
Medium ($800–$1999 per fortnight)	33 (41.3)		85 (48.0)	
High ($2000 or more per fortnight)	36 (45.0)		69 (39.0)	
SEIFA IRSAD quintiles		0.744		0.569
Q1—Most disadvantaged	26 (32.5)		88 (49.7)	
Q2	20 (25.0)		51 (28.8)	
Q3	15 (18.7)		24 (13.6)	
Q4	11 (13.8)		11 (6.2)	
Q5—Most advantaged	8 (10.0)		3 (1.7)	

Level of significance: *p* < 0.05 *, *p* < 0.01 **, *p* < 0.001 ***.

**Table 4:** Bivariate relationship between explanatory variables and bullying victimization by remoteness—Child data.

	Bullying Victimization—Child Data			
	Major Cities (*n* = 113)		Rural/Remote (*n* = 227)	
	*n* (%)	*p*-Value	*n* (%)	*p*-Value
Dental problems		0.027 *		0.054
No	63 (55.7)		137 (60.4)	
Yes	50 (44.3)		90 (39.6)	
Age		<0.001 ***		<0.001 ***
10 to £12	90 (79.7)		161 (70.9)	
≥13 to 15	23 (20.3)		66 (29.1)	
Gender		0.217		0.602
Boys	60 (53.1)		111 (48.9)	
Girls	53 (46.9)		116 (51.1)	
School Grade		0.080		0.110
Grade 4	0 (0.0)		4 (1.8)	
Grade 5	45 (39.8)		80 (35.2)	
Grade 6	44 (38.9)		65 (28.6)	
Grade 7	2 (1.8)		14 (6.2)	
Grade 8	15 (13.3)		46 (20.3)	
Grade 9	7 (6.2)		18 (7.9)	
School type		0.812		0.233
Private	10 (8.8)		8 (3.5)	
Catholic	16 (14.2)		17 (7.5)	
Government	87 (77.0)		202 (89.0)	
Parents’ educational qualification		0.762		0.010
Year 12 or below	46 (40.7)		104 (45.8)	
Certificate III or IV	34 (30.1))		88 (38.8)	
Diploma or above	33 (29.2)		35 (15.4)	
Parents’ employment status		0.261		0.980
Unemployed	44 (38.9)		100 (44.1)	
Employed	69 (61.1)		127 (55.9)	
Parents’ happiness with life		0.029 *		0.203
Unhappy	15 (13.3)		12 (5.3)	
Neither	17 (15.0)		24 (10.6)	
Happy	81 (71.7)		191 (84.1)	
Community safety		0.166		0.034 *
Safe	101 (89.4)		202 (89.0)	
Unsafe	12 (10.6)		25 (11.0)	
Child oral hygiene practice		0.648		0.015 *
Once a day or more	95 (84.1)		176 (77.5)	
Rarely or never	18 (15.9)		51 (22.5)	
Family functioning		0.057		0.646
Fair/Poor	5 (4.4)		7 (3.1)	
Very good/Good	108 (95.6)		220 (96.9)	
Family experiences racism		0.278		0.224
No	48 (42.5)		123 (54.2)	
Yes	65 (57.5)		104 (45.8)	
Family income		0.842		0.684
Low ($0–$799 per fortnight)	10 (8.9)		26 (11.5)	
Medium ($800–$1999 per fortnight)	46 (40.7)		111 (48.9)	
High ($2000 or more per fortnight)	57 (50.4)		90 (39.6)	
SEIFA IRSAD quintiles		0.189		0.121
Q1—Most disadvantaged	41 (36.3)		117 (51.5)	
Q2	30 (26.5)		63 (27.8)	
Q3	19 (16.8)		23 (10.1)	
Q4	15 (13.3)		16 (7.1)	
Q5—Most advantaged	8 (7.1)		8 (3.5)	

Level of significance: *p* < 0.05 *, *p* < 0.01 **, *p* < 0.001 ***.

**Table 5:** Logistic models for bullying victimization.

	Parent Data		Child Data	
	Major Cities	Rural/Remote	Major Cities	Rural/Remote
	aOR (95% CI)	aOR (95% CI)	aOR (95% CI)	aOR (95% CI)
Dental problems (ref. No)				
Yes	2.20 ** (1.20, 4.02)	1.41 (0.93, 2.14)	1.57 (0.87, 2.83)	1.38 (0.94, 2.04)
Age (ref. 10 to ≤12 years)				
≥13 to 15	1.40 (0.76, 2.56)	0.82 (0.54, 1.24)	0.19 *** (0.10, 0.36)	0.42 *** (0.29, 0.62)
Parents’ educational qualification (ref. Year 12 or below)				
Certificate III or IV	1.44 (0.72, 2.90)	1.65 * (1.06, 2.57)	1.09 (0.55, 2.16)	2.04 ** (1.35, 3.09)
Diploma or above	0.99 (0.48, 2.04)	1.35 (0.74, 2.45)	1.18 (0.58, 2.40)	1.56 (0.90, 2.70)
Parents’ happiness with life (ref. Happy)				
Neither	0.53 (0.15, 1.83)	1.54 (0.58, 4.07)	1.53 (0.41, 5.66)	2.27 (0.89, 5.76)
Unhappy	0.41 (0.15, 1.10)	1.24 (0.58, 2.66)	0.56 (0.21, 1.57)	2.08 * (0.99, 4.39)
Community safety (ref. Safe)				
Unsafe	2.67 (0.93, 7.59)	2.65 ** (1.30, 5.40)	2.26 (0.77, 6.62)	2.44 * (1.22, 4.86)
Child oral hygiene practice (ref. Once a day or more)				
Rarely or never	1.45 (0.63, 3.36)	1.33 (0.78, 2.27)	0.73 (0.31, 1.76)	2.33 ** (1.41, 3.86)
Family experiences racial discrimination (ref. No)				
Yes	3.27 *** (1.70, 6.29)	2.39 *** (1.58, 3.63)	0.69 (0.38, 1.26)	1.14 (0.78, 1.66)

aOR = Adjusted odds ratio; CI = confidence interval. Level of significance: *p* < 0.05 *, *p* < 0.01 **, *p* < 0.001 ***.

## Data Availability

The Footprints in Time—Longitudinal Study of Indigenous Children (LSIC) dataset is publicly available from the National Centre for Longitudinal Data (NCLD), Australian Data Archive (ADA) Dataverse upon request available at https://dataverse.ada.edu.au/dataverse/lsic (accessed on 3 September 2021). Authors do not have permission to share this unit record data without endorsement from the Australian Department of Social Services.
